# Isolation and characterization of a novel metagenomic enzyme capable of degrading bacterial phytotoxin toxoflavin

**DOI:** 10.1371/journal.pone.0183893

**Published:** 2018-01-02

**Authors:** Ji-Eun Choi, Cuong Mai Nguyen, Boyoung Lee, Ji Hyun Park, Joon Young Oh, Jung Sup Choi, Jin-Cheol Kim, Jae Kwang Song

**Affiliations:** 1 Research Center for Bio-based Chemistry, Korea Research Institute of Chemical Technology (KRICT), Daejeon, Republic of Korea; 2 Research Center for Eco-Friendly New Materials, Korea Research Institute of Chemical Technology (KRICT), Daejeon, Republic of Korea; 3 Department of Phytochemistry, Vietnam Institute of Industrial Chemistry, HoanKiem, Hanoi, Vietnam; 4 Department of Agricultural Chemistry, Institute of Environmentally Friendly Agriculture, College of Agriculture and Life Sciences, Chonnam National University, Gwangju, Republic of Korea; 5 Department of Green Chemistry and Environmental Biotechnology, Korea University of Science and Technology (UST), Daejeon, Republic of Korea; Dong-A University, REPUBLIC OF KOREA

## Abstract

Toxoflavin, a 7-azapteridine phytotoxin produced by the bacterial pathogens such as *Burkholderia glumae* and *Burkholderia gladioli*, has been known as one of the key virulence factors in crop diseases. Because the toxoflavin had an antibacterial activity, a metagenomic *E*. *coli* clone capable of growing well in the presence of toxoflavin (30 μg/ml) was isolated and the first metagenome-derived toxoflavin-degrading enzyme, TxeA of 140 amino acid residues, was identified from the positive *E*. *coli* clone. The conserved amino acids for metal-binding and extradiol dioxygenase activity, Glu-12, His-8 and Glu-130, were revealed by the sequence analysis of TxeA. The optimum conditions for toxoflavin degradation were evaluated with the TxeA purified in *E*. *coli*. Toxoflavin was totally degraded at an initial toxoflavin concentration of 100 μg/ml and at pH 5.0 in the presence of Mn^2+^, dithiothreitol and oxygen. The final degradation products of toxoflavin and methyltoxoflavin were fully identified by MS and NMR as triazines. Therefore, we suggested that the new metagenomic enzyme, TxeA, provided the clue to applying the new metagenomic enzyme to resistance development of crop plants to toxoflavin-mediated disease as well as to biocatalysis for Baeyer-Villiger type oxidation.

## Introduction

The 7-azapteridine antibiotics, toxoflavin (1,6-dimethylpyrimido[5,4-*e*]-1,2,4-triazine-5,7(1*H*,6*H*)-dione) and its analogs such as methyltoxoflavin, fervenulin and reumycin, belong to the prosthetic group of a bacterial yellow pigment that is highly toxic to plants, fungi, animals, humans and microorganisms. Toxoflavin was first isolated from *Pseudomonas cocovenenans*, with bongkrekic acid as two toxins that caused food poisoning [1, 2]. Toxoflavin is naturally synthesized by several bacteria such as *Burkholderia glumae*, *Burkholderia plantarii*, and *Burkholderia gladioli*. Toxoflavin was known to be one of the critical virulence factors in crop diseases such as bacterial panicle blight, also known as seedling rot or grain rot, which is caused by *B*. *glumae* and *B*. *gladioli* [3, 4]. Toxicity of bacterial toxoflavin as a major virulence factor has been assigned to its ability to function as an active electron carrier between NADH and oxygen. The action of toxoflavin bypasses the cellular cytochrome system and causes the generation of hydrogen peroxide in the presence of oxygen and light [5, 6]. This may also explain why toxoflavin shows antibacterial, antifungal and herbicidal activities [7].

Infection of toxoflavin-producing bacteria, particularly seed-borne *B*. *glumae*, has led to severe losses in rice crops in the world [6, 8, 9]. Bacterial grain rot occurring at the flowering stage of rice under high degree of temperature and moisture caused a significant loss in the rice yield up to 34%. In the southern USA, outbreaks of bacterial grain rot in rice fields in Louisiana in 1995 and 1998 made the rice yield loss up to 40% [4]. Currently, there are few control methods available for bacterial panicle blight of rice. Oxolinic acid treatment to rice seed was highly effective for the control of rice disease, but *B*. *glumae* strains naturally resistant to oxolinic acid have already occurred [10]. Although some varieties of rice exhibit partial resistance to bacterial panicle blight, no rice variety showing complete resistance to the disease is currently available [9, 11].

There has been growing interest both in interference with biosynthesis and in degradation of toxoflavin. An effective enzyme to degrade the toxin itself can be used to serve as an anti-virulence strategy in toxoflavin-mediated diseases of rice. Recently, *Paenibacillus polymyxa* strain JH2 that survived in the presence of toxoflavin was isolated from rice seed samples [12]. From this strain, a toxoflavin-degrading enzyme (TflA) capable of degrading toxoflavin was identified. The TflA enzyme catalyzed the aromatic ring-opening reaction by incorporating two atoms of dioxygen in toxoflavin and required Mn^2+^ ion and dithiothreitol, unlike other extradiol dioxygenases [13–15]. Nowadays the metagenomic DNA library consisting of genomes from as-yet uncultured or uncultivable microorganisms plays an important role in the identification of new enzymes with unique properties [16, 17]. In this study, we first attempted to discover a new toxoflavin-degrading enzyme from metagenome. A high-throughput screening process to detect the toxoflavin degradation activity from metagenome was successfully carried out. After expression of the metagenomic gene and purification of enzyme, toxoflavin degradation reaction by the metagenome-derived toxoflavin-degrading enzyme was further characterized.

## Materials and methods

### High-throughput screening for toxoflavin-degrading activity from metagenomic DNA library

The metagenome libraries used to screen toxoflavin-degrading activity were from Korea National Research Resource Bank (http://www.knrrc.or.kr/). The glycerol stock of metagenomic *E*. *coli* pools were stored at -70°C in a deep freezer. Each *E*. *coli* pool was thawed and inoculated into 800 μL of Luria-Bertani broth supplemented with toxoflavin (20 μg/mL) and antibiotics depending on types of plasmids and fosmids (34 μg chloramphenicol/mL, 50 μg ampicillin/mL and 10 μg tetracycline/mL) in deep-well plates. The deep-well plates were placed in a microplate incubator (Heidoph, Germany) and cultured at 37°C for 44 h in a shaking incubator. After 20 and 40 h cultivation, 100 μL of the cultures were transferred into 96 well plates to measure optical density (OD) at 600 nm. After the initial screening, metagenomic pools which displayed OD values over than 0.2 were spread on LB agar plates to separate the positive metagenomic pool into individual *E*. *coli* colonies. Each of clones were inoculated again into 800 μL of LB medium supplemented with toxoflavin (20 μg/mL) and ampicillin (50 μg/mL). To avoid the isolation of false-positive clones, several clones showing growth at 20 μg/mL of toxoflavin were successively transferred to fresh LB medium containing 20, 30, and 40 μg/mL of toxoflavin and further incubated for each 2 day. Finally, several *E*. *coli* clones carrying toxoflavin-degrading activity in the presence of toxoflavin were identified by measuring OD values and subjected for further genetic analysis.

### Genetic analysis

The toxoflavin-degrading clones were cultivated in LB medium for isolation of plasmid DNA using QIAGEN Plasmid Mini kit (Qiagen, Germany). The sequences of inserted DNA were determined with universal sequencing primer M13F (5′-GTAAAACGACGGCCAGT-3′) and M13R (5′-GCGGATAACAATTTCACACAGG-3′) using the automatic DNA analyzer (Applied biosystem, USA) at the Macrogen sequencing service (Macrogen, Daejeon, Korea). The obtained sequences were retrieved for sequence comparisons from protein and nucleotide database on the NCBI server and ORF finder (http://www.ncbi.nlm.nih.gov/Entrez/). Sequence similarity searches were performed with the BLASTX program and then homologous sequences were aligned with ClustalW (http://www.ebi.ac.uk/Tools/msa/clustalw2/) and MegAlign program (DNAstar, USA). Based on the sequencing results, a toxoflavin-degrading fosmid was selected and named as M2-67-B11.

A restriction enzyme map of M2-67-B11 fosmid was constructed by digestion of middle and end of each putative open reading frame (ORF) with restriction enzymes, SbfI, SacI, PstI, and AvaI. To find the ORF responsible for toxoflavin degradation, each DNA fragment was ligated into pUC19 vector (NEB, England) and then the recombinant plasmids were introduced into *E*.*coli* XL1-Blue cell (RBC, Taiwan). Each transformant was cultured in LB medium containing toxoflavin of 30 μg/mL and measured the optical density at 600 nm. The plasmid containing ORF related toxoflavin-degrading activity was named as pUC-TxeA. In addition, the deduced amino acid sequences of the selected ORF, *txeA*, were analyzed.

### Expression and purification of toxoflavin-degrading enzyme

The plasmid pUC-TxeA was amplified by the polymerase chain reaction (PCR) with two primers (ToxF, 5′-aaaGGATCCatgaatcagcctcctcc-3′ and ToxR, 5′-aaaGGTACCgccgcgacgtacccaca-3′), which contained BamHI and KpnI restriction sites, respectively. The PCR mixtures contained 50 ng of the pUC-TxeA plasmid as the initial template, 250 ng of each primer, 200 μM of each dNTP, and 5 U of Taq DNA polymerase in Taq DNA polymerase reaction buffer. The PCR started with a denaturation step at 95°C for 30 sec, followed by 30 cycles of amplification (30 sec at 95°C, 30 sec at 55°C, and 1 min at 72°C), and a final extension step at 72°C for 10 min. The PCR products purified with the QIAquick PCR Purification Kit (Qiagen) were digested with BamHI and KpnI and ligated into pQE30 vector. The recombinant plasmid was introduced into *E*.*coli* JM109, and the transformant was grown in LB broth until the optical density reached to 0.6 at 600 nm. Induction was done by adding 0.5 mM of isopropyl β-D-1-thiogalactopyranoside (IPTG) with shaking at 18°C. After 20 h, the cells were harvested using a centrifuge at 4000 rpm and 4°C for 30 min. The cell pellet was washed twice with distilled water and homogenized in Tris-HCl buffer (pH 8.0, 50 mM) using an ultrasonicator (Sonics and Materials, USA) followed by centrifugation at 10,000 rpm for 30 min. The supernatant was applied onto the column for the 6X histidine tag-based affinity chromatography. The unbound proteins were removed by 50 mM sodium phosphate buffer (pH 8.0) containing 300 mM NaCl and 20 mM imidazole. The protein bound to the column was then eluted by 50 mM sodium phosphate buffer containing 300 mM NaCl with 250 mM imidazole. The eluted sample was dialyzed in 20 mM sodium acetate buffer (pH 4.5) and kept at 4°C.

### Enzymatic properties of toxoflavin-degrading enzyme

Enzymatic properties of the purified TxeA, such as the optimum pH and temperature, and metal ion preferences, was investigated using high performance liquid chromatography (HPLC) and thin layer chromatography (TLC)-based methods. Unless otherwise notes, all reactions were performed under 180 rpm and 25°C for 60 min in 800 μL of reaction mixture containing 500 μL of sodium citrate buffer (20 mM, pH 5), 100 μL of dithiothreitol (DTT), 100 μL of MnCl_2_ (0.1M), 50 μL of enzyme stock solution (0.4 mg/ml) and 50 μL of toxoflavin (0.64 mg/mL; Sigma-Aldrich). The reaction was quenched by addition of 800 μL of 10 M urea followed by filtration through a pall protein concentrator (10,000 kDa cut off) at 7,500 rpm and 30°C for 15 min. To determine the enzymatic thermostability, the residual activity was measured after TxeA was preincubated at different temperatures (15–50°C) for 60 min. The effect of pH on catalytic activity was examined in either 20 mM sodium citrate buffer (pH 4.0–5.5), or 20 mM sodium phosphate buffer (pH 6.0–8.0). Toxoflavin remaining in the reaction solution was analyzed by HPLC as described below. The effect of metal ions on toxoflavin-degrading activity was tested with 100 mM of metal ions including MnCl_2_, FeCl_2_, CuCl_2_, CoCl_2_, CaCl_2,_ and MgCl_2_.

### Analytical methods

The concentration of residual toxoflavin and the enzymatic product in reaction solutions were analyzed using HPLC (Waters 996 PDA HPLC system; Waters Corp., Milford, MA, USA) equipped with a diode array detector. Briefly, a 150 mm × 4.6 mm Phenomenex Luna C18 column (particle size of 5 μm) was used to separate the toxoflavin at a flow rate of 1 mL/min. The solvents constituting the mobile phase were 100% water (solvent A, containing 0.1% acetic acid) and 100% MeOH (solvent B, containing 0.1% acetic acid). The gradient program was as follows: 0–20 min, 10% B; 20–40 min, linear gradient solvent system from 10% B to 100% B; 40–43 min, 100% B. The retention time of toxoflavin was 6.1 min.

### Large-scale purification of degradation products of toxoflavin and its analogs

A large-scale reaction was carried out with a total of 120 ml reaction mixture consisting of 85 mL sodium citrate buffer (20 mM, pH 5.0), 15 mL DTT (100 mM), 7.5 mL MnCl_2_ (100 mM), 7.5 mL toxoflavin (1.6 mg/mL) and 5 mL enzyme solution (0.5 mg/mL). The reaction mixture was incubated at 20°C for 1 h in a shaking incubator (200 rpm) and then filtrated with an ultrafiltration spin filter (molecular weight cut-off of 10 kDa). The filtered reaction solution was applied onto C18 cartridge for solid phase extraction (Sep-Pak C18, Agilent Technology). The cartridge was rinsed successively with 150 mL water and 150 mL of 100% MeOH. The water fraction was again applied to the solid phase extraction process. The water fraction from the second extraction was freeze-dried to remove water and the residues were dissolved in 300 μL MeOH. The enzymatic product was purified by preparative TLC (Silica Gel 60 F254 plates, Merck) using solvent system of chloroform and methanol (8:2, v/v). To identify the products, TLC was performed in a chamber containing mixed solvents of chloroform and methanol (8:2, v/v) at room temperature.

### Determination of final products

The chemical structures of the final degradation products of toxoflavin and its analogs were determined mainly by mass spectrometry (MS), proton and carbon nuclear magnetic resonance (^1^H-NMR, ^13^C-NMR) analyses. Electrospray ionization-mass spectra (ESI-MS) of final products were recorded on a single quadrupole mass spectrometer equipped with an ESI (MSD1100, Hewlett-Packard Co., CA). The ^1^H- and ^13^C-NMR spectra were recorded in CD_3_OD (Merck) on a Bruker AMX-500 (500 MHz) spectrometer (Bruker Analytische Messtechnik GmbH, Germany) at 500 MHz for ^1^H-NMR spectra and 125 MHz for ^13^C-NMR spectra. The spectra were referenced to either tetramethylsilane (TMS) or solvent signals.

## Results and discussion

### Screening for toxoflavin-neutralizing activity from the metagenomic DNA library

Toxoflavin appears to act as an antibiotic against *E*. *coli* by acting as an electron carrier which makes possible a by-passing of the cytochrome-system. It has been known that toxoflavin completely inhibited the growth of *E*. *coli* in concentrations of less than 3 μg/ml [5]. We also confirmed that the *E*. *coli* host cells containing a metagenomics DNA could not grow in LB medium supplemented with toxoflavin at the final concentration of 20 μg/ml. By cultivating *E*. *coli* libraries harboring the metagenomics DNA library in the presence of toxoflavin, it was therefore intended to discover a metagenome-derived enzyme activity capable of neutralizing the antibiotic effect of toxoflavin.

The metagenomic DNA libraries from soil samples were constructed in *E*. *coli* and then about 1,173,000 *E*. *coli* clones were screened for an enzyme activity neutralizing the growth inhibition effect of toxoflavin toward *E*. *coli* (S1 Fig). A single pool of independent *E*. *coli* clones (M2-67) was identified on the basis of a difference in OD value at 600 nm (OD_600nm_) between the cultures with or without toxoflavin (20 μg/ml). The M2-67 pool showed an increase of more than 0.2 in OD_600nm_, indicating that an *E*. *coli* clone of the pool exhibit toxoflavin-degrading or resistance activity.

**Fig 1:**
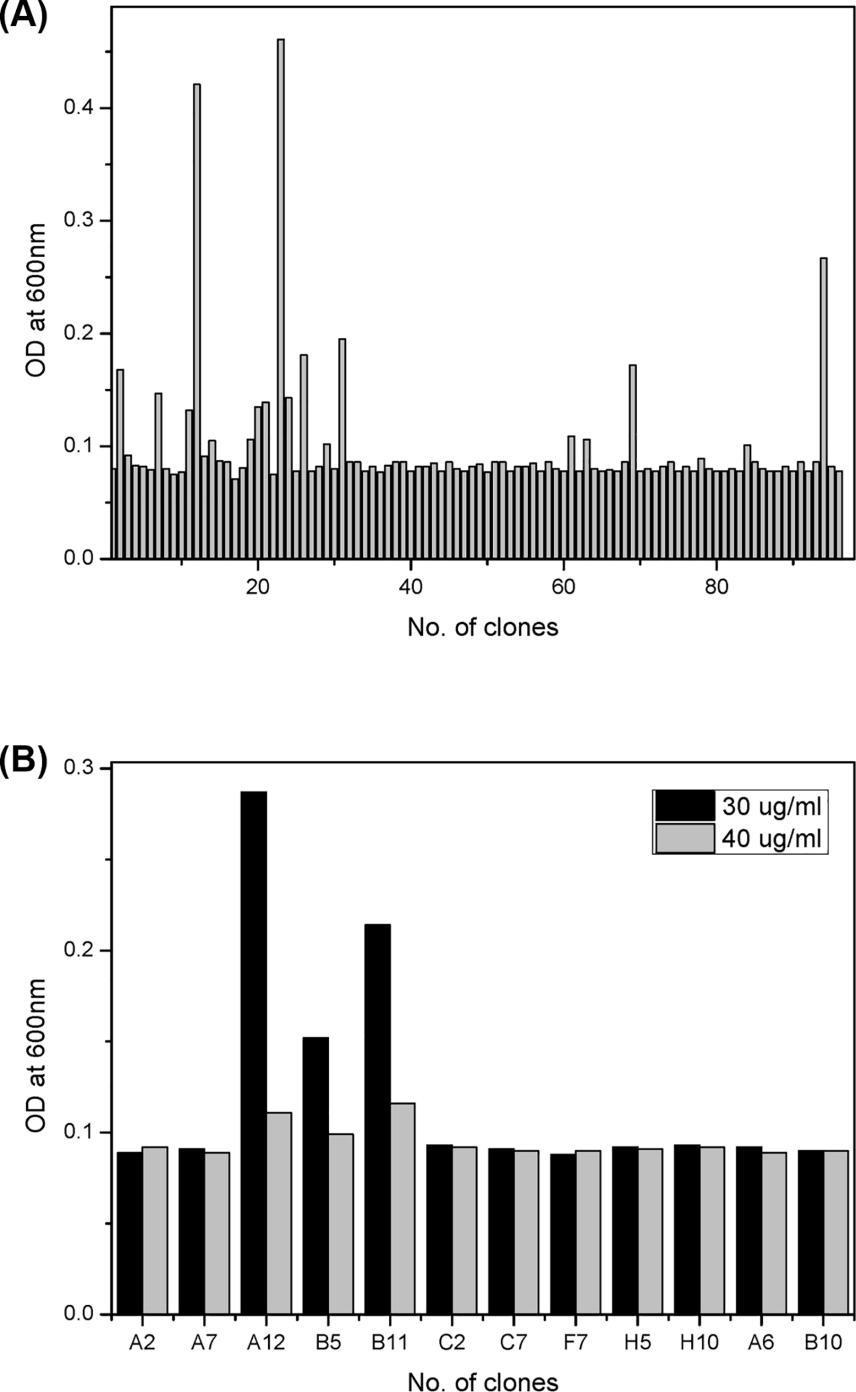
Metagenomic library screening for toxoflavin-degrading activity. The metagenome pool M2-67 was spread on LB agar plates and each single colony was inoculated in deep-well plate containing LB medium supplemented with 20 μg/mL toxoflavin. After incubation for 2 days, OD_600nm_ values of each cell culture were measured (A). Twelve metagenomic *E*. *coli* clones showing cell growth over 0.1 of OD_600nm_ in the presence of 20 μg/mL toxoflavin were further examined in LB medium containing 30 and 40 μg/mL toxoflavin (B).

About 3,000 *E*. *coli* clones from the M2-67 pool were developed on agar plates and each colony was transferred to LB medium containing 20 μg/mL toxoflavin. Twelve *E*. *coli* clones showing a relatively higher increase in cell growth (Fig 1A) were selected and then the growth inhibition effect was examined by increasing the final concentration of toxoflavin to 30 and 40 μg/mL (Fig 1B). Three *E*. *coli* clones (M2-67-A12, M2-67-B5, and M2-67-B11) exhibited a distinctive cell growth at 30 μg/mL toxoflavin, although the cell growth of all the *E*. *coli* clones was almost suppressed at 40 μg/mL toxoflavin. They were finally selected as an *E*. *coli* clone harboring a new metagenomic toxoflavin-neutralizing activity.

### Sequence and structural analysis of toxoflavin-degrading enzyme

DNA sequencing analysis revealed that the three *E*. *coli* clones had the same metagenomic DNA fragment and originated from three independent colonies of the single *E*. *coli* clone. The metagenomic insert DNA was about 1.7 kb and expected to include four ORF (S2 Fig). To identify a gene responsible for toxoflavin degradation, the several restriction enzyme-digested fragments were subcloned and the cell growth inhibition of *E*. *coli* was examined in the presence of toxoflavin (30 μg/ml). Only *E*. *coli* clone harboring a putative glyoxalase/bleomycin resistance protein-encoding orf1 showed the cell growth.

The gene conferring the toxoflavin-neutralizing activity was named *txeA* (GenBank accession no. KT210132) and encoded 140 amino acid residues, which was much shorter than the previously reported toxoflavin lyase-encoding gene (*tflA*) encoding 221 amino acid residues [13, 14]. They showed very low sequence identity (less than 10%) in their amino acid sequences. The new metagenomic enzyme, TxeA, was most similar only to the putative genes derived from whole-genome sequencing studies (S1 Table).

**Fig 2:**
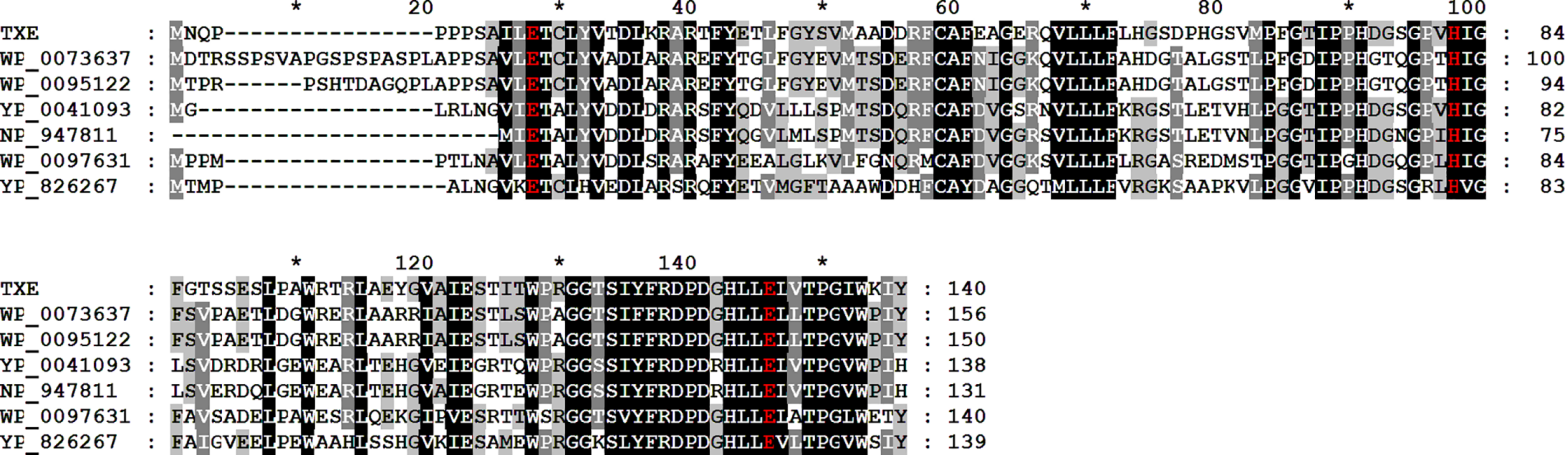
The comparison of amino acid sequences of TxeA and its six most homologous sequences. The amino sequences of homologs were identified by a BLAST search of the amino acid sequence of TxeA and the corresponding GenBank accession numbers of the sequences are as follows: Glyoxalase from *Opitutaceae bacterium* TAV1, WP_007363792.1; Glyoxalase from *Opitutaceae bacterium* TAV5; glyoxalase/bleomycin resistance protein/dioxygenase from *Rhodopseudomonas palustris* DX-1, YP_004109357.1; lactoylglutathionelyase from *Rhodopseudomonas palustris* CGA009, NP_947811.1; Glyoxalase from *Microvirga* sp. WSM3557, WP_009763194.1; glyoxalase/bleomycin resistance protein/dioxygenase from *Candidatus Solibacter usitatus* Ellin6076, YP_826267.1. Identical and similar amino acid sequences are shaded in black and gray, respectively. The putative catalytic residues were shown as red characters.

On the basis of the conserved domain analysis using the Conserved Domain Database (http://www.ncbi.nlm.nih.gov/Structure/cdd/cdd.shtml), TxeA was expected as a new member of the vicinal oxygen chelate (VOC) metalloenzyme superfamily which included the bleomycin resistance protein, glyoxalase I and type I ring-cleaving dioxygenases. The toxoflavin biosynthetic gene cluster found in the genome sequences of *Pseudomonas protegens* Pf-5 [18] contained ToxM of 137 amino acids (GenBank accession no. AAY90317), a monooxygenase that degraded toxoflavin [19], whereas the first well-characterized cluster of *Burkholderia glumae* did not contained a toxoflavin-degrading enzyme. ToxM was also revealed to belong to the glyoxalase/bleomycin resistance protein/dioxygenase superfamily (Pfam 00903), which was one of the VOC superfamily. Based on sequence analysis, TxeA was therefore supposed as a metalloprotein with extradiol dioxygenase activity. The VOC enzyme superfamily requires an enzyme-bound metal ion and thus shares the conserved metal-binding motif including either two histidines and one glutamate or one histidine and two glutamates [20] (S3 Fig). The conserved amino acids in metal-binding motif were found in the metagenomic TxeA at Glu-12, His-82 and Glu-130 (Fig 2) and in *Pseudomonas protegens* Pf-5 ToxM at Glu-9, His-79 and Glu-127 [19] (S4 Fig). Likewise, TflA was reported to have three manganese-binding ligands at His-60, Glu-113, and Glu-138 [13, 14].

Although the whole sequences in putative metal binding sites of TxeA were not exactly matched with TflA, both enzymes showed the same variation of the 2-His-1-carboxylate facial triad. The 2-His-1-carboxylate facial triad is a common structural motif of the active sites in a number of mononuclear non-heme Fe(II) enzymes [13]. According to the structural analysis, the metal binding site of TflA was surrounded by funnel-like space which enclosed by the hydrophobic residues, including Phe-94, Pro-95, Phe-96, Phe-97, Ile-111, Leu-170, Phe-172, Leu-181, Trp-189, and Leu-190 [13]. In the case of TxeA, the aromatic and hydrophobic residues were also existed around the putative metal binding residues, His-83 and Glu-130. In particular, Trp-111, Pro-113, Trp-136, and Leu-128 of TxeA were located at the similar position with Phe-94, Pro-95, Phe-96, and Ile-111 of TflA, respectively, which played an important role in positioning the incoming toxoflavin into the productive binding mode for catalysis [14]. However, the study of three-dimensional conformation is further required to prove the above prediction.

TxeA was analyzed by native PAGE and gel filtration analysis to test the possibility of oligomer. However, we could not find any evidence that TxeA existed as oligomeric structure. Therefore, TxeA has very unique characteristics in term of having much shorter amino acid residues than TflA. To reveal the mechanisms of toxin-degrading process, the study of three-dimensional conformation is further required. Nevertheless, it is suggested that the differences of metal binding sites suggested multiple evolutionary paths involving gene fusion, domain swapping, circular permutation, and individual amino acid mutations [13].

### Biochemical characterization of TxeA

**Fig 3:**
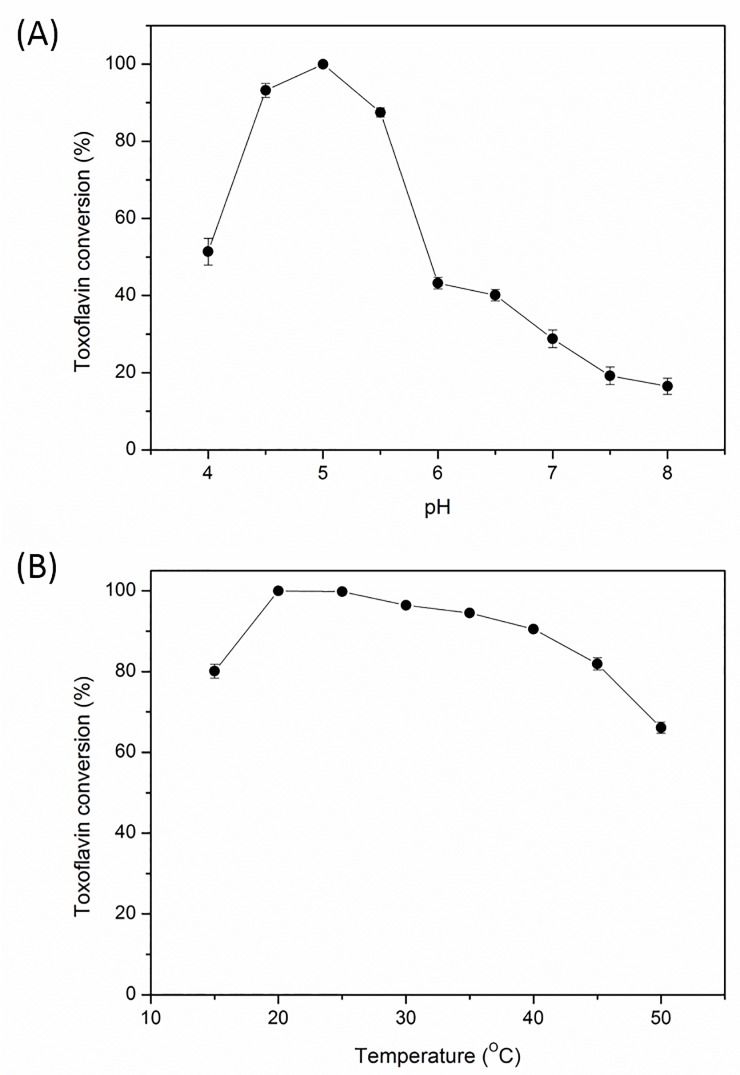
Effect of pH and temperature on toxoflavin degradation reaction: (A) effect of pH; (B) effect of temperature.

The 6X histidine-tagged TxeA was highly purified and appeared as a strong single band (S5 Fig). To determine the optimum pH for toxoflavin reaction, the serial pH was evaluated from 4–8. The toxoflavin conversion increased and reached 100% until pH 5 and then dramatically decreased when pH higher than pH 5.5 (Fig 3A). The peak area of toxoflavin degradation product also reached a maximum value at pH 5. The optimal pH for toxoflavin degradation reaction is 5.0, which was significantly different from TflA [12]; TflA showed no toxoflavin degradation activity at pH lower than 5.5. In comparison, the TflA enzyme exhibited the highest activity at pH 7.0 and 6.5, respectively, and could fully degrade toxoflavin at pH higher than 6 [12–15]. However, our TxeA reached toxoflavin conversion lower than 45% at pH higher than 6.0.

Catalytic activity for toxoflavin degradation on broad range of temperature was tested at an enzyme concentration of 1.875 μg/mL. TxeA showed maximum activity at 20°C (Fig 3B). In addition, the toxoflavin conversion rate was markedly high between 20 and 40°C, retaining at least 90% of the maximum activity at 40°C. The enzyme activity was maintained at 15 and 45°C nearly 80% of maximum conversion rate, however it severely decreased at temperatures more than 45°C. Besides, slight increase in by-products production at 25°C were observed in HPLC analysis, the result suggests that TxeA showed a good toxoflavin degrading activity at broad range of temperature of 20–40°C. In comparison, TflA showed optimal temperature at 25°C, and furthermore the toxoflavin degradation activity was very low at 20°C [12]. The initial reaction velocities of the enzyme obeyed Michaelis-Menten model (S6 Fig). The values of turnover number (*k*_cat_), apparent binding constants of substrate (*K*_M_) and catalytic efficiency (*k*_cat_/*K*_M_*)* for TxeA were determined to be 1.57 s^-1^, 1.4 x 10^−5^ M and 1.12 x 10^5^ s^-1^ M^-1^ at 20°C and pH 5.0, respectively. The kinetic parameters of TxeA exhibited moderate catalytic efficiency as the average enzyme exhibits a *k*_cat_/*K*_M_ of ~10^5^ s^-1^ M^-1^ [21]. While substrate turnover of TxeA was not fast, the *K*_M_ value of TxeA was very low when compared with the distributions of k_*cat*_ and *K*_M_ values of several thousand enzymes collected from the literature [21], indicating that TxeA had an extremely high binding affinity for toxoflavin.

**Fig 4:**
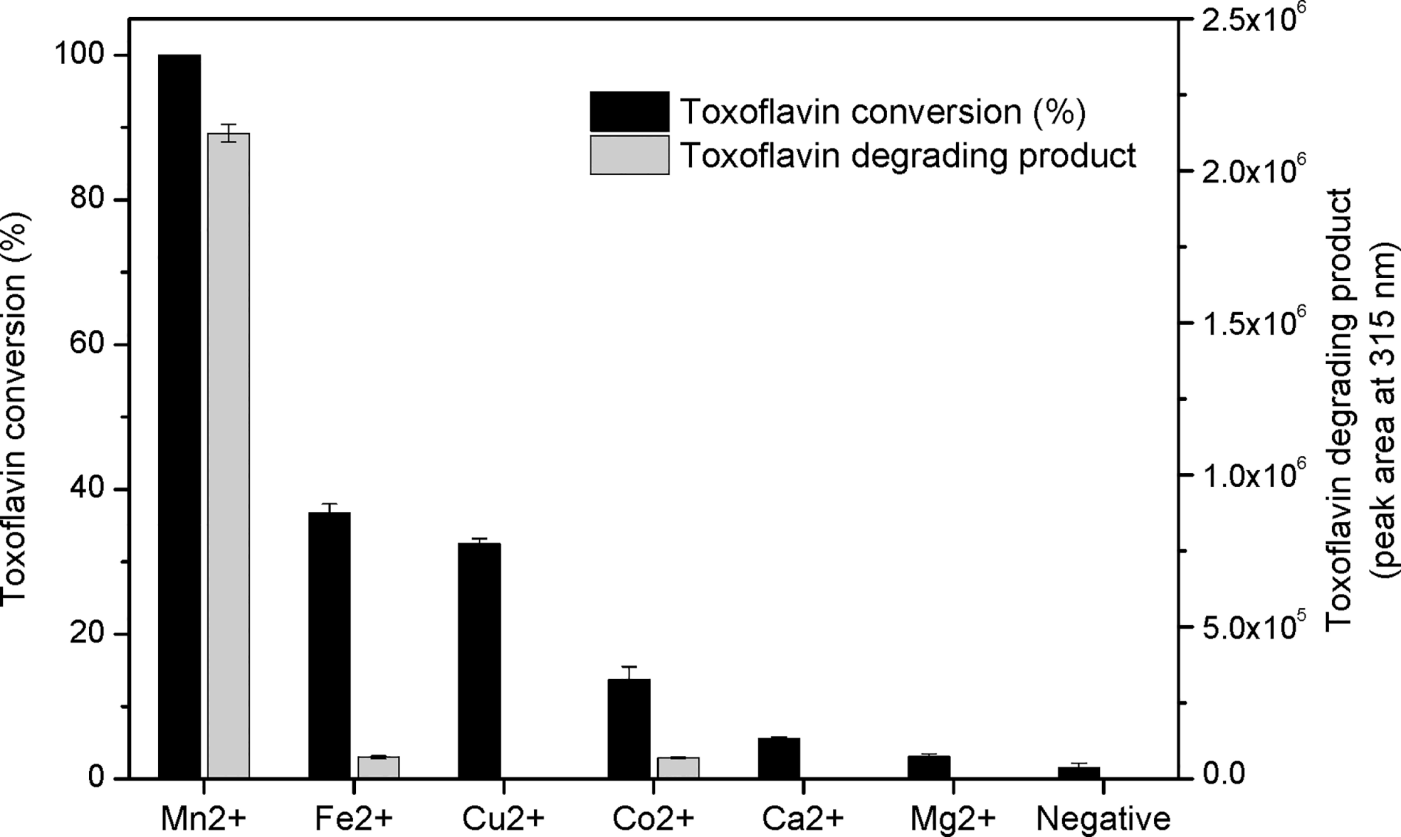
Effect of Ion metal (X^2+^) on toxoflavin degradation reaction.

In the agreement with TflA of *P*. *polymyxa*, the enzymatic conversion of toxoflavin by TxeA required oxygen in the presence of Mn^2+^ and DTT. In this study, TxeA agreed with previous results, which showed high degrading activity in presence of 100 mM of Mn^2+^ (Fig 4). On the contrary, addition of metal ions to the enzyme resulted in low activity for Fe^2+^ (30%) and Co^2+^ (12.48%) and no activity for Cu^2+^, Mg^2+^ and Ca^2+^. Even though toxoflavin concentration was decreased, no or little enzymatic product was found when Fe^2+^, Cu^2+^, and Co^2+^ were added. In this case, TXF may be degraded following the way of natural degradation which was also enhanced in the presence of Fe^2+^, Cu^2+^, and Co^2+^. TflA showed very low activity in presence with Fe^2+^ and no activity in presence with Fe^3+^, Co^2+^, Cu^+^,Cu^2+^ and Ba^2+^.^9^ This results indicated that TxeA and TflA was specific for only Mn^2+^ ion for toxoflavin degrading rather than any other divalent cations. VOC superfamily is a group of structurally related proteins that provide a metal coordination environment with two or three open or readily accessible coordination sites to promote direct electrophilic participation of the metal ion in catalysis [19]. Extradiol dioxygenases, the functional member of VOC superfamily, were known to Mn^2+^and Fe^2+^-dependent enzymes. Because the redox potential of the metal center needs to be matched with that of the substrates, it is supposed that Mn^2+^-dependent enzymes such as extradiol dioxygenase will not function with Fe^2+^.

### Determination of the final products of toxoflavin and its derivatives

**Fig 5:**
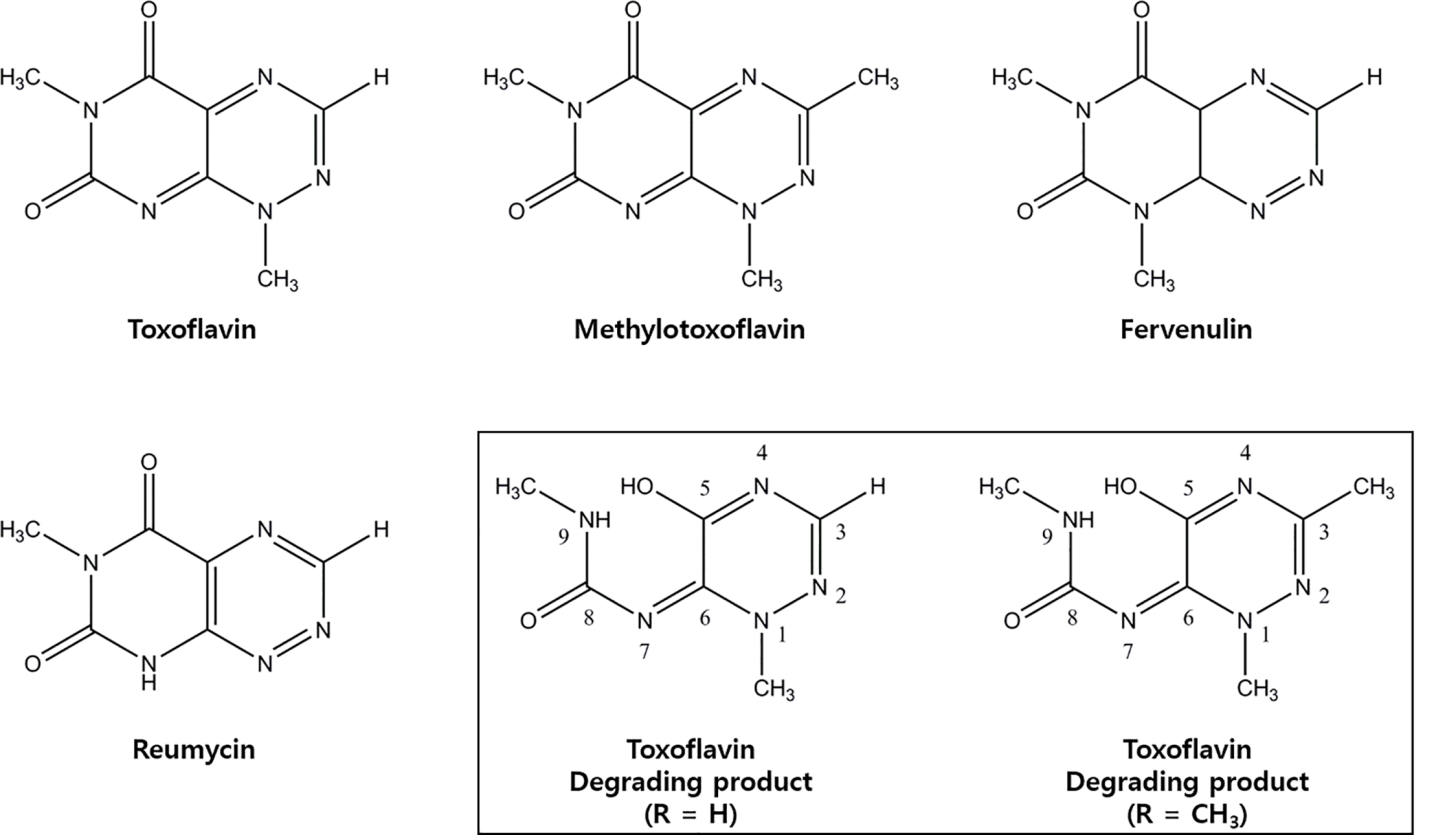
Chemical structures of toxoflavin, methyl toxoflavin, fervenulin and reumycin and enzymatic products.

The final product obtained from enzyme reactions using toxoflavin and its derivatives including methyltoxoflavin, fervenulin, and reumycin were analyzed by LC-ESI (Fig 5 and S7 Fig). The final product of toxoflavin degradation showed a base peak at *m/z* 184.2 which was assigned as the [M + H^+^]^+^ ion. In addition, the [M + Na^+^]^+^ and [2M + Na^+^]^+^ ions were also observed at *m/z* 206.2 and 389.3, respectively (S8A and S8B Fig). ^1^H-NMR analysis indicated five signals including 2 methyls, 1 methine, 1 NH, and 1 OH (S9 Fig). In the ^13^C-NMR spectrum, six signals could be observed (S10 Fig). The ESI analysis of converted product from methyltoxoflavin also revealed the presence of ions with *m/z* of 198.2, 221.2 and 417.4, as shown in S8C and S8D Fig. The *m/z* 198.2 was the most abundant ion in the spectrum and assigned as the [M + H^+^]^+^ion, and the others was assigned as [M + Na^+^]^+^ and [2M + Na^+^]^+^, respectively. The ^1^H-NMR clearly revealed five signals including 3 methyls, 1 NH, and 1 OH (S11 Fig), and in the ^13^C-NMR spectrum, seventh signals could be observed and assigned (S11 Fig). These results indicated that one carbon atom from methyltoxoflavin was lost during its conversion to final product under TxeA catalysis. The final product was (1*E*)-1-(5-hydroxy-1,3-dimethyl-1,2,4-triazin-6(1*H*)-ylidene)-3-methylurea, a triazine. Even though toxoflavin and methyltoxoflavin could be completely degraded, reumycin and fervenulin were slightly degraded under the catalysis of TxeA at a concentration of 40 μg/ml, 20–25% and 5–10%, respectively.

The final products obtained from toxoflavin and methyltoxoflavin suggested that the mechanism of enzymatic reaction of TxeA (S13 Fig) could be the same as those proposed for TflA [13, 15]. According to the suggestions, toxoflavin or methyltoxoflavin is reduced into dihydrotoxoflavin or dihydromethyltoxoflavin form in the presence of DTT. Previously, the reduced form of toxoflavin was readily bound to and acted upon by TflA. Furthermore, the reduced product was unstable and rapidly re-oxidized to toxoflavin so that the reaction was initiated by addition of DTT rather than TflA. In this study, we also confirmed that toxoflavin was not degraded without DTT. Therefore, we could suggest that reduction of toxoflavin was required for further reaction of both TxeA and TflA. The study introducing a mechanism for toxoflavin degradation proposed the Baeyer-Villiger oxidation to produce triazine (S13 Fig) [15]. In addition, the ToxM reaction products identified by UV spectra, MS and MS/MS fragmentation patterns were same as those of TflA. Interestingly, the final products of toxoflavin conversion using three different enzymes, TxeA, TflA and ToxM, were identical. The sequence identities between enzymes (44%; TxeA and ToxM, 10%; TxeA and TflA) were very low and moreover, the length of amino acids of TxeA and ToxM were much shorter than TflA (S4 Fig). The only common aspects of three enzymes were existence of conserved metal binding regions; therefore we suggested that specific amino acid residues including three metal binding sites were critical for toxoflavin degradation.

## Conclusions

It has become increasingly important to develop better methods of disease control for grain rot, seedling rot and bacterial panicle blight in rice. In this study, a new enzyme capable of degrading toxoflavin, the major virulence factor in the bacterial infection of rice, could be identified by screening metagenomic libraries for resistance to growth inhibitory activity of toxoflavin against the host *E*. *coli* cells. The toxoflavin-degrading enzyme, TxeA, was the first metagenome-derived enzyme unique in amino acid sequences and biochemical properties such as high activity in a broad temperature range and under acidic conditions, as compared with the toxoflavin-degrading TflA from *Paenibacillus polymyxa* strain JH2 capable of surviving in the presence of toxoflavin. Homology-based modeling of 3D structure and identification of enzymatic degradation products also suggested that the new metagenomic enzyme could be used for biocatalytic Baeyer-Villiger type oxidation. This study also demonstrated that the metagenomic approach is very useful for expanding our knowledge of enzyme diversity, especially for bacterial phytotoxin-degrading enzymes.

## Supporting information

S1 Fig The screening of metagenomic library for searching a toxoflavin degrading clone. (PDF)

S2 Fig A restriction map of 1.7 kb fragment of metagenome clone, M2-67-11. (PDF)

S3 Fig Sequence alignment of TxeA, TflA, and VOC superfamily proteins. (PDF)

S4 Fig Amino acid sequence alignment of three toxoflavin-degrading enzymes. (PDF)

S5 Fig SDS-PAGE analysis of the purified TxeA. (PDF)

S6 Fig Evaluation of Michaelis-Menten constant and maximum reaction rate. (PDF)

S7 Fig HPLC analysis of toxoflavin (315 nm), methyltoxoflavin (254 nm) fervenulin (254 nm) and reumycin (260 nm) degradation. (PDF)

S8 Fig LC-MS chromatography of enzymatic degradation. (PDF)

S9 Fig 1H-NMR of degrading products. (PDF)

S10 Fig 13C-NMR of degrading products. (PDF)

S11 Fig 1H-NMR of degrading products of methyltoxoflavin. (PDF)

S12 Fig 13C-NMR of degrading product of methyltoxoflavin. (PDF)

S13 Fig Mechanism for toxoflavin degradation by TxeA. (PDF)

S1 Table Homologous proteins of TxeA. (PDF)
