# Supplementary material for: Isolation and characterization of a novel metagenomic enzyme capable of degrading bacterial phytotoxin toxoflavin
Source: PLoS One. 2018 Jan 2;13(1):e0183893. doi: 10.1371/journal.pone.0183893 (PMC5749703; doi:10.1371/journal.pone.0183893)
Supplement: S1 Fig — (PDF) [file pone.0183893.s001.pdf]

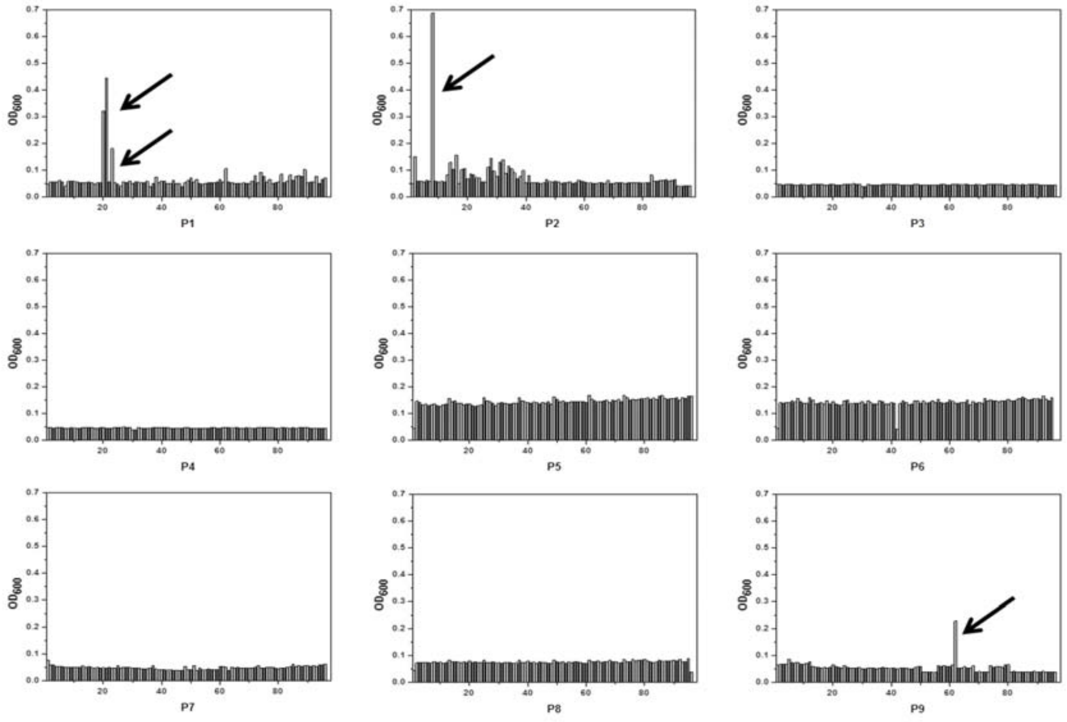

S1 Fig. The screening of metagenomic library for searching a toxoflavin degrading clone. Metagenomics pools grown in 9 deep well plates were further incubated in LB medium containing 20 μg/mL toxoflavin. Based on optical densities showing over OD_{600nm} 0.2, 5 metagenomic pools containing about 3,000 clones were selected.
