# Supplementary material for: Isolation and characterization of a novel metagenomic enzyme capable of degrading bacterial phytotoxin toxoflavin
Source: PLoS One. 2018 Jan 2;13(1):e0183893. doi: 10.1371/journal.pone.0183893 (PMC5749703; doi:10.1371/journal.pone.0183893)
Supplement: S2 Fig — (PDF) [file pone.0183893.s002.pdf]

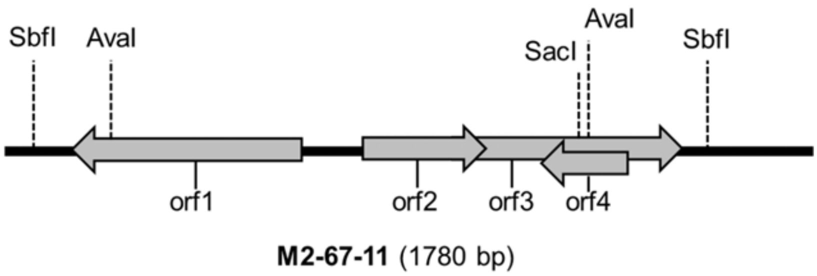

S2 Fig. A restriction map of 1.7 kb fragment of metagenome clone, M2-67-11. Putative ORFs shown on the restriction map were inferred from sequence analysis. The names of the putative ORFs are as follows: orf1, Glyoxalase/bleomycin resistance protein; orf2, Nickel dependent hydrogenase; orf3, oxidoreductase; orf4, hypothetical protein. The restriction sites of SbfI, SacI, PstI, and AvaI are indicated on the map.
