# Supplementary material for: Isolation and characterization of a novel metagenomic enzyme capable of degrading bacterial phytotoxin toxoflavin
Source: PLoS One. 2018 Jan 2;13(1):e0183893. doi: 10.1371/journal.pone.0183893 (PMC5749703; doi:10.1371/journal.pone.0183893)
Supplement: S3 Fig — (PDF) [file pone.0183893.s003.pdf]

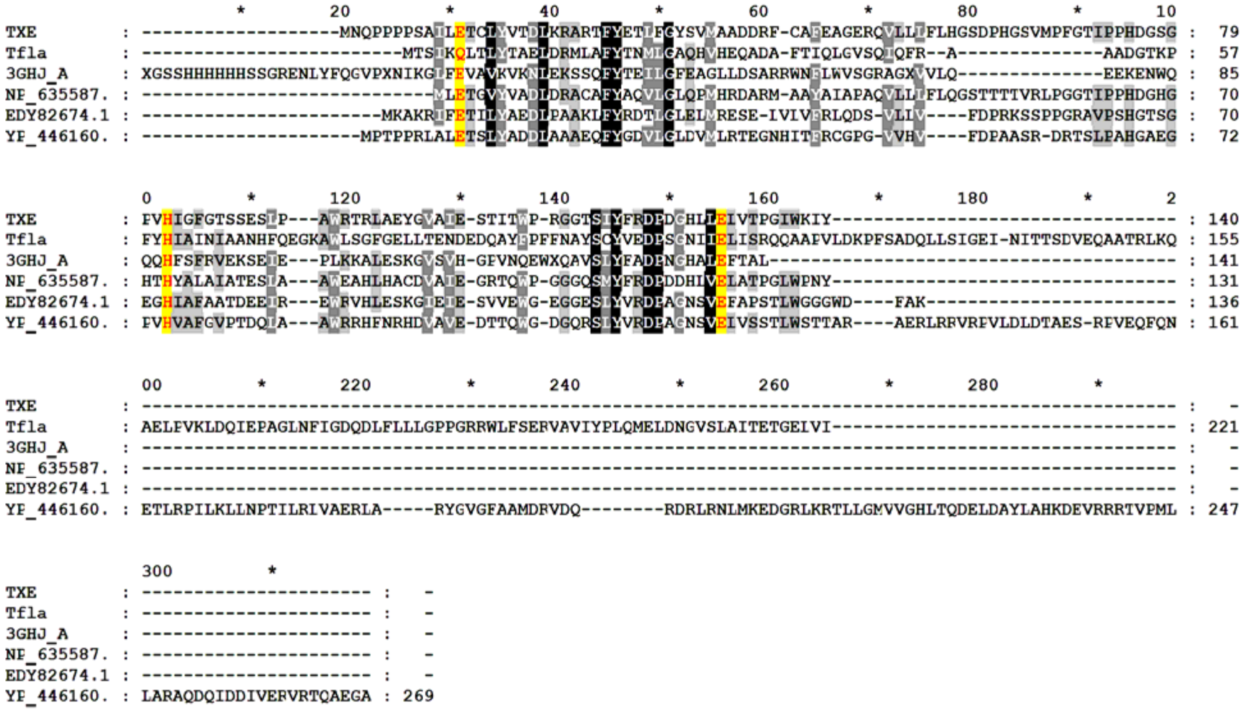

S3 Fig. Sequence alignment of TxeA, TflA, and VOC superfamily proteins. The amino acid sequence of VOC superfamily proteins and the corresponding GenBank accession numbers are as follows: 3GHJ_A; Chain A, Crystal Structure From The metagenomic protein from Halifax harbour sewage outfall, NP_635587; lactoyl glutathione lyase from *Xanthomonas campestris* pv.; glyoxalase family protein from *Verrucomicrobiae bacterium* DG1235, YP_446160; glyoxalase family protein from *Salinibacterruber* DSM 13855. The highlighted red letters indicate the conserved amino acids in metal-binding motif.
