# Supplementary material for: Isolation and characterization of a novel metagenomic enzyme capable of degrading bacterial phytotoxin toxoflavin
Source: PLoS One. 2018 Jan 2;13(1):e0183893. doi: 10.1371/journal.pone.0183893 (PMC5749703; doi:10.1371/journal.pone.0183893)
Supplement: S4 Fig — (PDF) [file pone.0183893.s004.pdf]

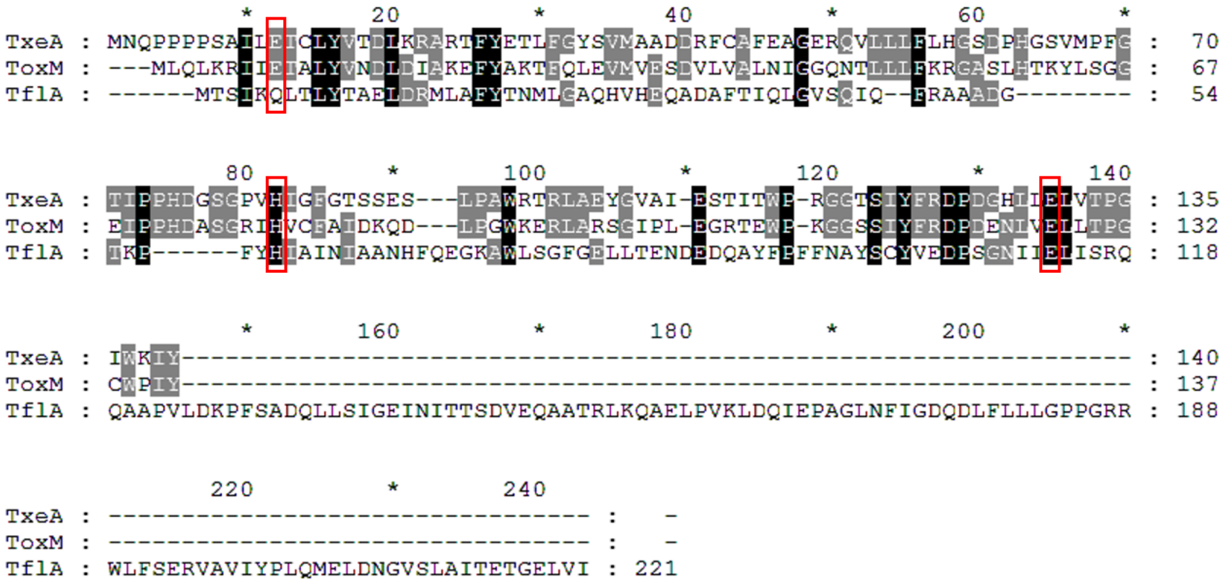

S4 Fig. Amino acid sequence alignment of three toxoflavin-degrading enzymes. TxeA (GenBank accession no. KT210132) obtained from the metagenome in this study, ToxM (GenBank accession no. AAY90317) identified from the genome sequence of *Pseudomonas protegens* Pf-5 and TflA (GenBank accession no. ADK47414) cloned from *Paenibacillus polymyxa* JH2 are compared. The red squares indicate the conserved amino acids in metal-binding motif.
