# Supplementary material for: Isolation and characterization of a novel metagenomic enzyme capable of degrading bacterial phytotoxin toxoflavin
Source: PLoS One. 2018 Jan 2;13(1):e0183893. doi: 10.1371/journal.pone.0183893 (PMC5749703; doi:10.1371/journal.pone.0183893)
Supplement: S5 Fig — (PDF) [file pone.0183893.s005.pdf]

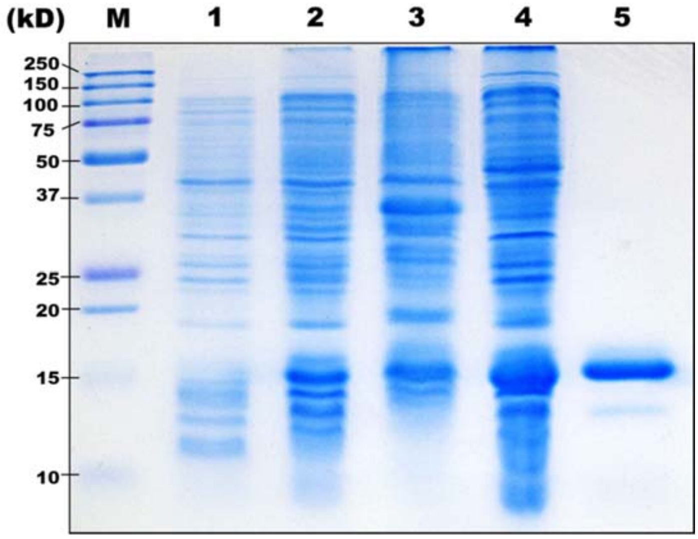

S5 Fig. SDS-PAGE analysis of the purified TxeA. Lane M; molecular weight standards, Lane 1; total cellular extracts before induction, Lane 2; total cellular extracts after induction, Lane 3; insoluble fraction of cellular extracts, Lane 4; soluble fraction of cellular extracts, Lane 5; purified TxeA.
