# Supplementary material for: Isolation and characterization of a novel metagenomic enzyme capable of degrading bacterial phytotoxin toxoflavin
Source: PLoS One. 2018 Jan 2;13(1):e0183893. doi: 10.1371/journal.pone.0183893 (PMC5749703; doi:10.1371/journal.pone.0183893)
Supplement: S6 Fig — (PDF) [file pone.0183893.s006.pdf]

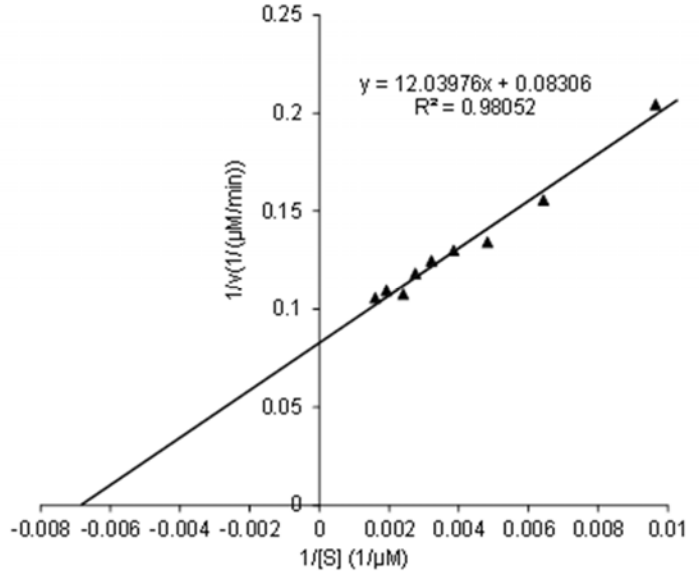

S6 Fig. Evaluation of Michaelis-Menten constant and maximum reaction rate. Total enzyme concentration used for enzyme kinetics was 0.1275 μM.
