# Supplementary material for: Isolation and characterization of a novel metagenomic enzyme capable of degrading bacterial phytotoxin toxoflavin
Source: PLoS One. 2018 Jan 2;13(1):e0183893. doi: 10.1371/journal.pone.0183893 (PMC5749703; doi:10.1371/journal.pone.0183893)
Supplement: S7 Fig — (PDF) [file pone.0183893.s007.pdf]

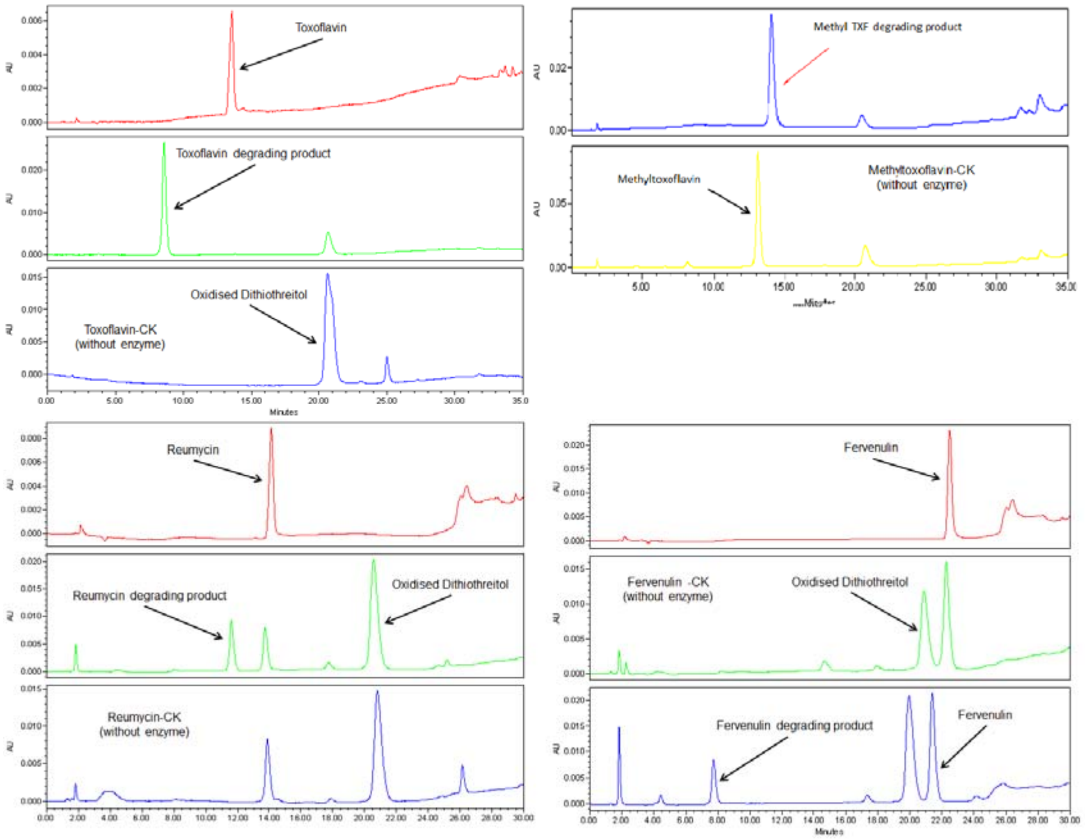

S7 Fig. HPLC analysis of toxoflavin (315 nm), methyltoxoflavin (254 nm) fervenulin (254 nm) and reumycin (260 nm) degradation. The purified TxeA of 40 µg/ml was used.
