# Supplementary material for: Isolation and characterization of a novel metagenomic enzyme capable of degrading bacterial phytotoxin toxoflavin
Source: PLoS One. 2018 Jan 2;13(1):e0183893. doi: 10.1371/journal.pone.0183893 (PMC5749703; doi:10.1371/journal.pone.0183893)
Supplement: S8 Fig — (PDF) [file pone.0183893.s008.pdf]

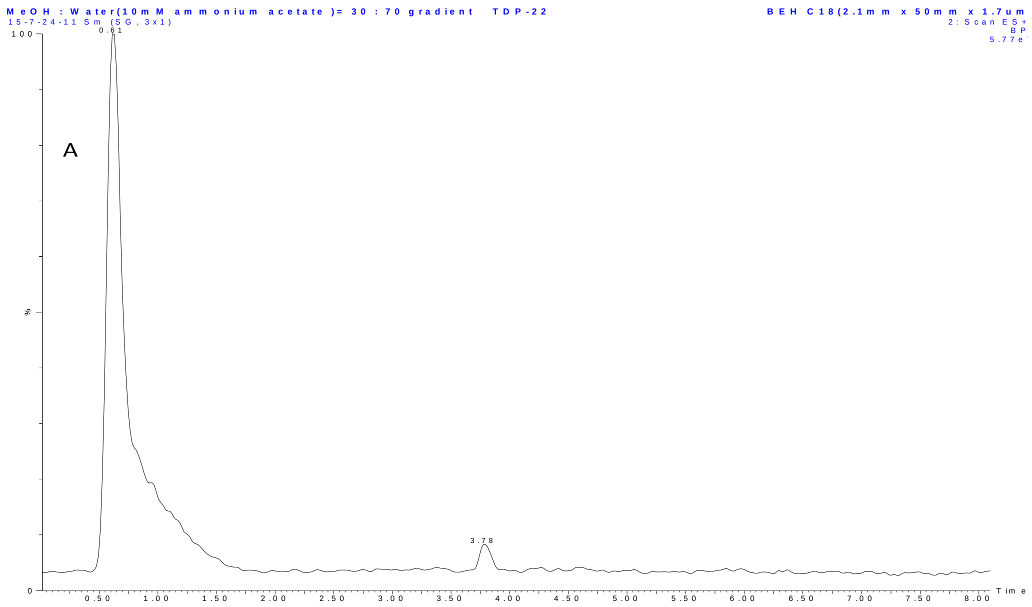
MeOH : Water(10mM ammonium acetate)= 30 : 70 gradient TDP-22
15-7-24-11 Sm (SG, 3x1)
BEH C18(2.1mm x 50mm x 1.7um
2: Scan ES+
BP
5.77e
A
0.61
3.78
100
%
0
0.50 1.00 1.50 2.00 2.50 3.00 3.50 4.00 4.50 5.00 5.50 6.00 6.50 7.00 7.50 8.00
Time

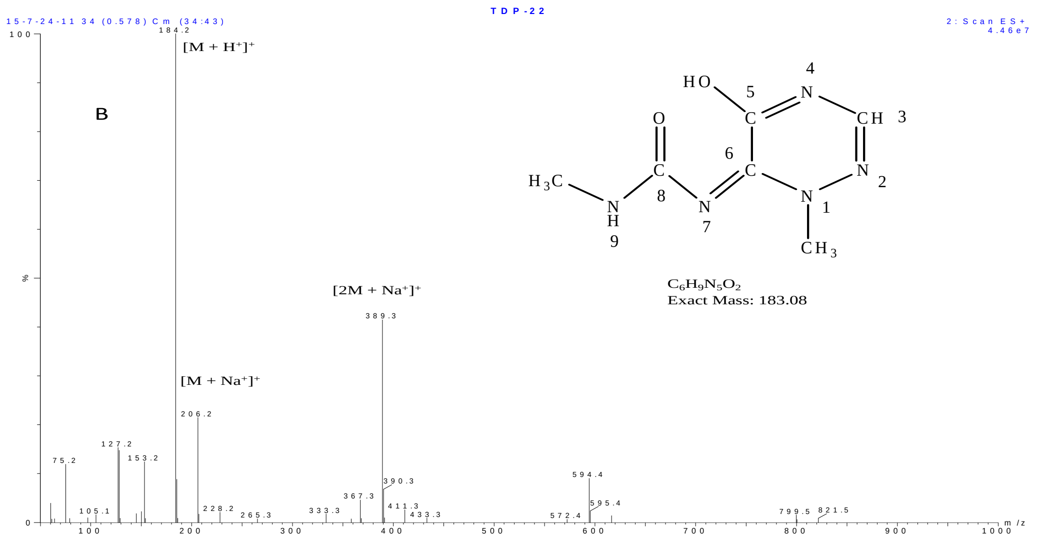
TDP-22
15-7-24-11 34 (0.578) Cm (34:43)
2: Scan ES+
4.46e7
B
184.2
[M + H⁺]⁺
[M + Na⁺]⁺
206.2
[2M + Na⁺]⁺
389.3
75.2
105.1
127.2
153.2
228.2
265.3
333.3
367.3
390.3
411.3
433.3
572.4
594.4
595.4
799.5
821.5
100
%
0
100 200 300 400 500 600 700 800 900 1000
m/z
HO
O
H3C
N
H
C
N
C
C
N
CH
N
N
CH3
1 2 3 4 5 6 7 8 9
C6H9N5O2
Exact Mass: 183.08

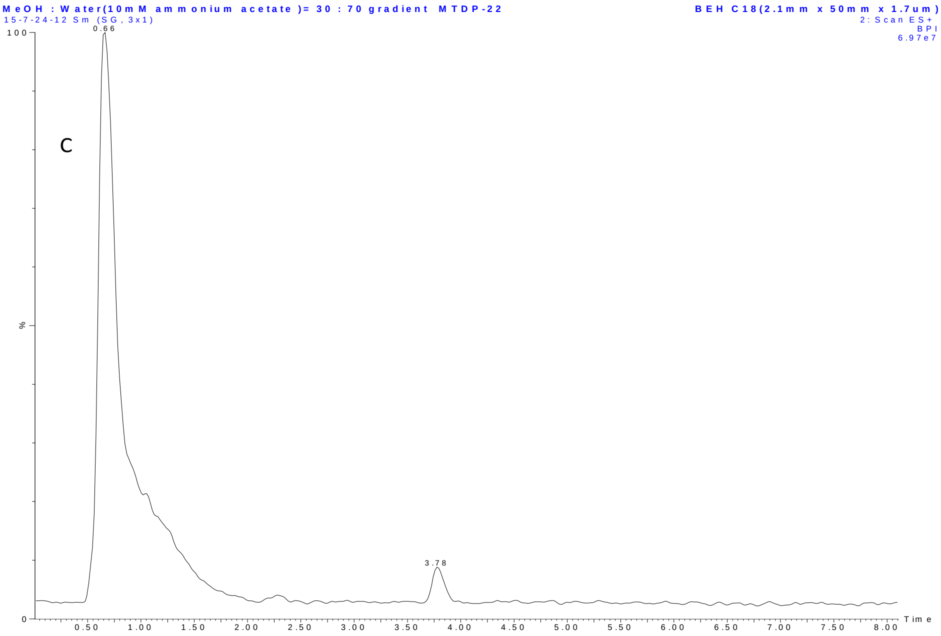

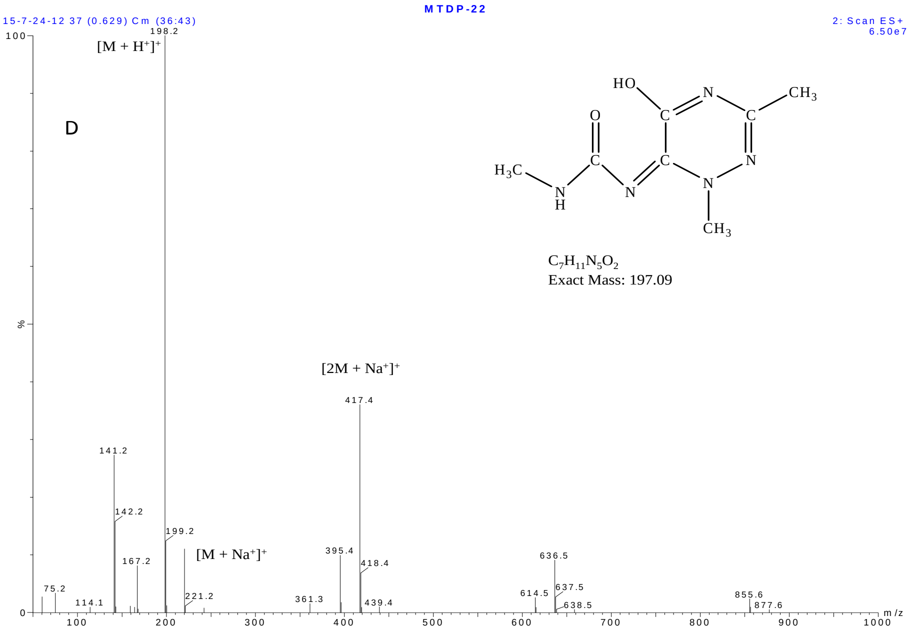

S8 Fig. LC-MS chromatography of enzymatic degradation of toxoflavin (A) and methyltoxoflavin (C). Mass spectrum of TDE catalyzed oxidation product of toxoflavin (R_f = 0.61 min) and methyltoxoflavin (0.623 min)
