# Supplementary material for: Isolation and characterization of a novel metagenomic enzyme capable of degrading bacterial phytotoxin toxoflavin
Source: PLoS One. 2018 Jan 2;13(1):e0183893. doi: 10.1371/journal.pone.0183893 (PMC5749703; doi:10.1371/journal.pone.0183893)
Supplement: S10 Fig — (PDF) [file pone.0183893.s010.pdf]

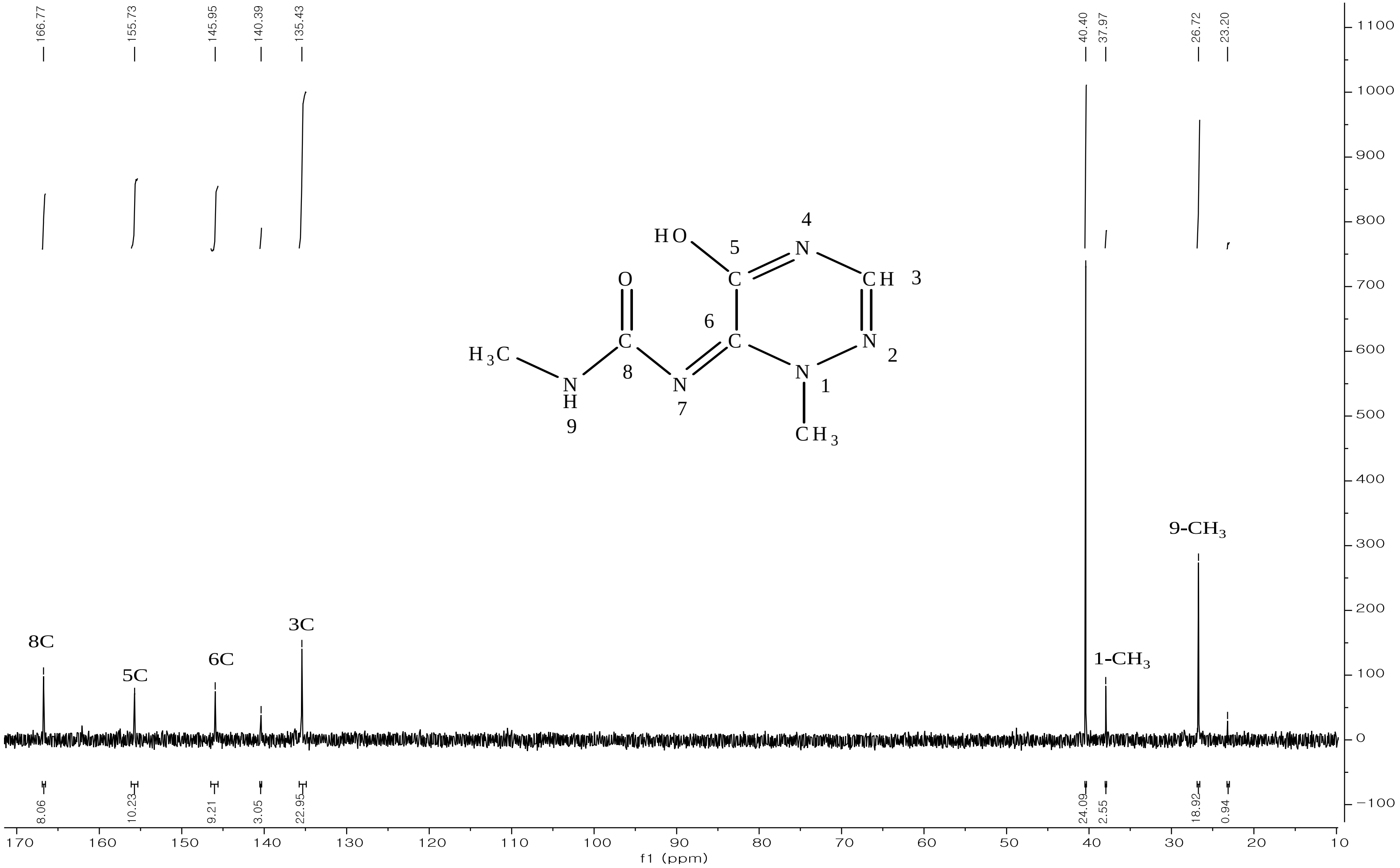

S10 Fig. ^{13}C-NMR of degrading products from TXE in D_2O. Insert shows peak assignments based on the final structure and enlargement of thespectrum.
