# Supplementary material for: Isolation and characterization of a novel metagenomic enzyme capable of degrading bacterial phytotoxin toxoflavin
Source: PLoS One. 2018 Jan 2;13(1):e0183893. doi: 10.1371/journal.pone.0183893 (PMC5749703; doi:10.1371/journal.pone.0183893)
Supplement: S13 Fig — (PDF) [file pone.0183893.s013.pdf]

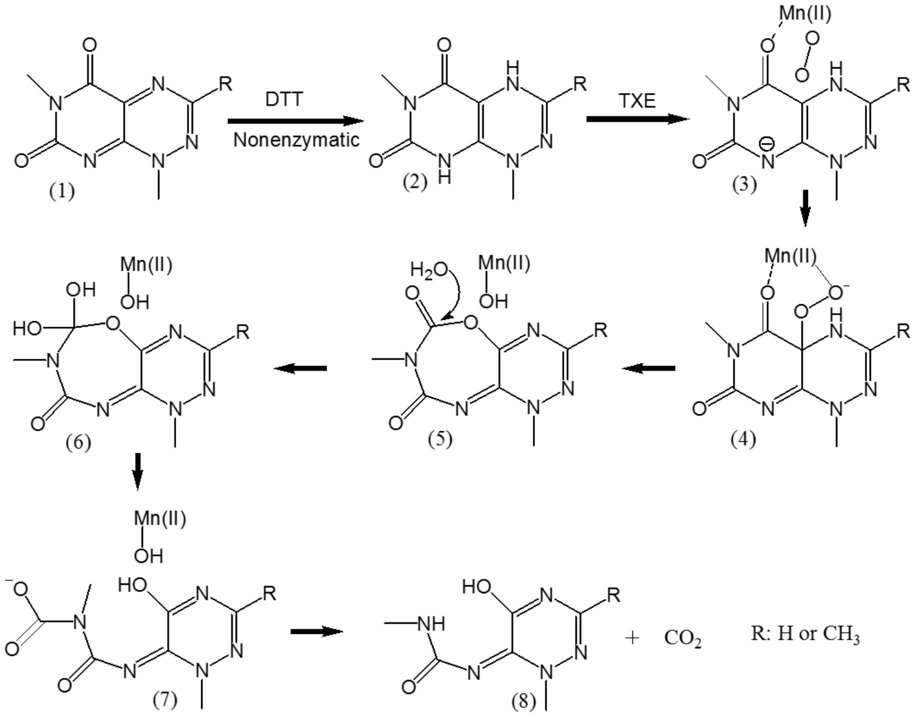

S13 Fig. Mechanism for toxoflavin degradation by TxeA. Toxoflavin or methyltoxoflavin (1) is reduced into dihydrotoxoflavin or dihydromethyltoxoflavin form (2), a substrate for the TxeA, in the presence of DTT. The compound (2) binds to enzyme (3) and then is oxidized to peroxide (4) before ring expanding via Baeyer-Villiger oxidation to yield (5) and $Mn(OH)_2$. The compound (5) is naturally hydrolyzed to (7) through tetrahedral intermediate (6) and then decarboxylated (7) to generate (8) and CO_2. The final products obtained from toxoflavin and methyltoxoflavin suggested that the mechanism of enzymatic reaction of TxeA (S14 Fig) could be the same as those proposed for TflA (Philmus B, Abdelwahed S, Williams HJ, Fenwick MK, Ealick SE, Begley TP. Identification of the product of toxoflavin lyase: degradation via a Baeyer–Villiger oxidation. J Am Chem Soc. 2012; 134:5326–5330).
