# Supplementary material for: Isolation and characterization of a novel metagenomic enzyme capable of degrading bacterial phytotoxin toxoflavin
Source: PLoS One. 2018 Jan 2;13(1):e0183893. doi: 10.1371/journal.pone.0183893 (PMC5749703; doi:10.1371/journal.pone.0183893)
Supplement: S1 Table — (PDF) [file pone.0183893.s014.pdf]

S1 Table. Homologous proteins of TxeA.

Description	Query cover	E value	Max ident	Accession
glyoxalase/bleomycin resistance protein/dioxygenase [*Opitutaceae bacterium* TAV5]	96%	3e-59	66%	ZP_09595956.1
lactoylglutathionelyase-like lyase [*Opitutaceae bacterium* TAV1]	93%	3e-56	65%	ZP_10268962.1
lactoylglutathionelyase-like lyase [*Microvirga sp.* WSM3557]	97%	3e-52	58%	ZP_10181559.1
glyoxalase/bleomycin resistance protein/dioxygenase [*CandidatusSolibacterusitatus* Ellin6076]	97%	3e-51	55%	YP_826267.1
glyoxalase/bleomycin resistance protein/dioxygenase [*Rhodopseudomonaspalustris* DX-1]	95%	2e-49	59%	YP_004109357.1
lactoylglutathionelyase [*Rhodopseudomonaspalustris* CGA009]	93%	6e-49	59%	NP_947811.1
